# Correction: Telemedicine via data glasses in CBRN protection suit—Evaluation of medical qualification and technical feasibility

**DOI:** 10.1371/journal.pone.0352425

**Published:** 2026-06-23

**Authors:** Sarah Bovenkerk, Anna Mueller, Rolf Rossaint, Michael Czaplik, Andreas Follmann

S1 Script is omitted from the list of Supporting Information. It can be viewed below.

## Supporting information

S1 Script Table 1. Script for the practical study procedure. (PDF)
