# Supplementary material for: Telemedicine via data glasses in CBRN protection suit—Evaluation of medical qualification and technical feasibility
Source: PLoS One. 2025 May 28;20(5):e0324558. doi: 10.1371/journal.pone.0324558 (PMC12118917; doi:10.1371/journal.pone.0324558)
Supplement: Attachment — Submitted filename: PONE-D-24-49405.docx [file pone.0324558.s001.docx]

PONE-D-24-49405

**Telemedicine via data glasses in CBRN portection suit – Evaluation of medical qualification and technical feasibility**

This is a well elaborated study that aims to examine technical feasibility of telemedicine in CBRN scenarios. However, I have some notes:

**INTRODUCTION/OBJECTIVES**

1- Title: *Telemedicine via data glasses in* ***CBRN*** *protection suit – Evaluation of medical qualification and technical feasibility*. Abbreviation CBRN. Its recommended define abbreviations clearly at first. Same for the abstract.

Line 21 (“Telemedicina via data glass in the Chemical, Biological, Radiological, and Nuclear (CBRN)”...)

2- Consider clarificate de objective in abstract: “data glasses was examined for its technical feasibility and the minimum required medical qualification”. Like line 99 “Examine the feasibility of data glasses in medical qualification...”

**METHODS**

3- What was the criteria to select de participants of the study?
4- Were there any other target parameters analysed under the x-ABCDE approach? The Authors analysed airways, breathing, circulation and mental status. Did the telemdicine equipment could help a less qualified professional placing cervical collar? (Since protecting spine is one of the first procedures in emergency setting)
5- Voice connection and volume and sound quality are essentials in telemedicine and figure 5 points diferences between equipements used in study. How to the Authors consider improve communication in CBRN scenarios that might interfer with telemedicine equipment (mainly radiological and nuclear accidents)?
6- Did the Authors consider placing others equipments that could support telemedicine in emergency setting (monitorization of the patient)? For example, using oximeter, sphygmomanometer to help the decision make by distance?
