# Supplementary material for: Telemedicine via data glasses in CBRN protection suit—Evaluation of medical qualification and technical feasibility
Source: PLoS One. 2025 May 28;20(5):e0324558. doi: 10.1371/journal.pone.0324558 (PMC12118917; doi:10.1371/journal.pone.0324558)
Supplement: Attachment — Submitted filename: Response to Reviewers.docx [file pone.0324558.s003.docx]

Response to Reviewers

Dear Sir or Madam,

thank you very much for taking the time to read my manuscript and for our comments and improvements. We have edited the manuscript accordingly and made the following changes:

Financial disclosure

The funding code of the study was incorrect. We request the following change in the submission portal. We have also added that the sponsor had no influence on the study with your proposal:

“Funded by the Federal Office of Civil Protection and Disaster Assistance as part of the TeleSAN project (FKZ: 41201/425). The sponsor had no influence on the study design, the data collection and analysis, the decision to publish or the preparation of the manuscript.“

Compensating interests

We request the following change in the submission portal:

“MC (CEO, co-founder) and RR (co-founder) are with Docs in Clouds TeleCare GmbH, a company that develops telemedicine software. This does not change our adherence to PLOS ONE policies on sharing data and materials. The other authors declare no conflicts of interest.”

Formatting

The formatting of the manuscript has been changed according to the PLOS ONE style guide.

Abstract

The word CBRN was written out to make it easier to understand for readers who only read the abstract.

The terms “hazmat suits” and “smart glasses” have been added to make it easier to understand in international context.

According to reviewer 2, we have changed two sentences in the abstract for better understanding.

Introduction

Drug therapies in emergency situations are severely restricted by non-medical emergency personnel. Two substances were listed as examples.

In the last section of the introduction, the current situation regarding the administration of medication by EMT-P and other non-medical emergency personnel in Germany was explained.

Reviewer 1 has noted that the last sentences of the introduction should be better moved to the conclusion. Due to the submissions guidelines of PLOS Onne, who would like a preview of the results in the introduction, it was decided not to postpone the two sentences.

Methods

Thank you for your comments on the methodology section. A detailed description of the questionnaires has been included to improve the presentation of the study design. As described, the study participants were selected in equal proportions according to their qualifications.

In addition, the information that both data glasses transmitted the sound in real time was added to the “Telemedicine” section.

The patient was monitored on site with the help of a surveillance monitor. The practical performance of vital sign measurements was not part of the study investigation, as all participants were appropriately trained.

Limitations

Due to the study design, a Hawthorne effect could not be ruled out with certainty. This aspect was included in the limitations. We have added the reference [17] for this purpose.

Discussion

According to Figure 5, the HMT-1 does not show poor audio quality, so it is not necessary to add a headset at this point. A headset could be used as a supplement for other glasses; this has been added accordingly in the text referring to Reviewer 2.

Reviewer 2 commented on whether additional medical equipment would be useful for telemedicine. As medical devices have to be cleaned or disposed of at great expense after use in contaminated areas, additional material is avoided wherever possible. In addition, a conceptual attempt is made to decontaminate the patient as quickly as possible. We have therefore dispensed with further equipment in the study and have not included this point in the paper. If you have any additional comments or requests for changes, please let us know.

The following point was included in the discussion: Despite the technical difficulties, it should not be ignored that the smart glasses have made life-saving measures possible in the first place, which would not be possible without telemedicine.

References

The references has been checked. Since reference [4] is no longer available, it was replaced by a reference with the same content.

A corresponding reference to the Hawthorne effect was added.

Tracking changes of the references is unfortunately not possible, so the changes in the references have been highlighted accordingly.
