# Supplementary material for: Correction: Telemedicine via data glasses in CBRN protection suit—Evaluation of medical qualification and technical feasibility
Source: PLoS One. 2026 Jun 23;21(6):e0352425. doi: 10.1371/journal.pone.0352425 (PMC13289856; doi:10.1371/journal.pone.0352425)
Supplement: S1 Script — (PDF) [file pone.0352425.s001.pdf]

# The script

### Table 1. Script for the practical study procedure.

| telemedicine physician | study participant | situation |
| --- | --- | --- |
| | | |
| Hello, this is the telemedicine physician of the city of cologne.<br><br>What can I do for you? | | The patient screams and moans in pain in the background |
| | answer study participant | |
| Okay, I can see the patient via the camera in your data glasses. If there are any problems, I'll let you know.<br><br>Look at the patient. Do you proceed according to the cABCDE scheme? Does the patient have an acute c-problem? So, is there a spurting bleed? | | |
| | answer study participant | |
| To stop the bleeding, you should apply a tourniquet to the patient. The tourniquet is in the emergency backpack.<br><br>Would you like me to explain again how to apply a tourniquet? | | |
| | The tourniquet is being prepared | |
| Briefly explain to the patient what you intend to do. First, place the tourniquet around the thigh and tighten the strap. Then turn the gag until the bleeding stops. Then turn the gag around again. | | |
| | The tourniquet ist applied. | |
| Has the bleeding stopped? | | Once the tourniquet is in place, the bleeding stops. |
| | The tourniquet ist beeing checked. | |
| If the tourniquet is not applied correctly or the test person has problems, the application is attempted again | | |
| Good, then check A. Is there an obvious A problem? Are the airways obstructed? | | Patient continues to scream and moan in pain in the background. |

| | | |
|---|---|---|
| | answer study participant | |
| And what about B? You could measure the oxygen saturation. | | |
| | $SpO_2$ measurement is prepared and performed. | |
| | | $Sp0_2$: 94% |
| Okay, no acute A or B problem. Is there a C problem? The monitor also shows a pulse via the Sp02 sensor. You can now also measure your blood pressure. | | |
| | pulse and blood pressure measurement | |
| | | pulse rate: 94 b/m<br>blood pressure: 94/60 mmHG<br>$SpO_2$: 92% |
| Okay, the patient is currently still stable. You continue with the scheme. Is there a D problem or E? Is the patient in pain? | | |
| | answer study participant | Patient continues to scream and moan in pain in the background. |
| Then you should give the patient a painkiller. The easiest way to do this is to administer the medication via the nasal mucous membranes. I'll explain how this works in a moment. Before you do this, you should disinfect both nostrils with cotton buds. Take a cotton bud for each nostril, disinfect in a circular motion and then put them to one side. | | |
| | The spot decontamination is performed | |
| Okay, now prepare the medication.<br>There is a bag with ampoules and syringes in your emergency backpack. You should give the patient 25mg Ketanest®. First you take a syringe, which is packed together with the nebulizer, and attach a | | |

| | | |
|---|---|---|
| cannula. Use the cannula to draw up 1 ml of the medication. This gives you 25mg. Discard the cannula in the waste. | | |
| | The analgesia is drawn up and prepared | |
| If you have already administered medication nasally, you can apply 1 ml of the medication to the patient's nostril.<br><br>If you have never administered a medication nasally before, I will explain the procedure to you again. | | |
| | answer study participant | |
| Okay, if you have air bubbles in the syringe, you can hold the syringe with the cone at an angle upwards and slowly push the plunger into the syringe. You should now have 1 ml of solution in the syringe. Then place the MAD applicator on the cone of the syringe. It looks similar to a white funnel and is also located in the small pocket. Then inject 1 ml of the solution into one nostril. | | |
| | drug administration via MAD. | |
| If there are any problems with the preparation and application of the medication, a new attempt is made | | |
| Now please put an FFP2 mask on the patient. | | |
| Then we re-evaluate again. Vital signs are stable? | | |
| | answer study participant | $SpO_2$: 68%<br>Pulse rate: 108 b/m<br>GCS: 6 |
| There is an acute A or B problem. The patient needs airway protection and respiratory support.<br>Have you ever placed a laryngeal mask? Then you can place a size 4 mask and try to ventilate the patient. | | |
| | answer study participant | |
| Take a size 4 laryngeal mask, the size is written on | | |

| | | |
|---|---|---|
| the back of the packaging. One end of the laryngeal mask has an opening with an elastic edge that can be blocked. A ventilation bag can be placed on the other end. Apply some lubricant to the blockable end. Then try to insert the laryngeal mask with the curved side into the mouth. This is not easy. At some point you will feel a kind of springy resistance. The position should then be correct. | | |
| | The laryngeal mask is placed and practical performance of ventilation | |
| If ventilation is not possible, the laryngeal mask is repositioned and ventilation is attempted again. | | |
| Okay. In order to stabilize the patient further, you can place another access line and connect an infusion.<br>To do this, you place an access in the patient's right lower leg bone.<br>To do this, take another 1-2 disinfectant wipes and dab the upper part of the lower leg over a large area | | |
| | spot decontamination | |
| Prepare an infusion. Take an infusion and connect and bleed a system. Then put the prepared infusion to one side. | | |
| | The infusion is being prepared | |
| If you have already placed an I.O. line once, you can place an I.O. line for the patient, connect the infusion and start it.<br>Otherwise I will explain the procedure to you. | | |
| | answer study participant | |
| There is also a bag with a bone drill and matching needles in the emergency backpack. Take a blue needle and put it on the drill. Then feel for a protrusion about 2-4 cm below the knee. Then drill vertically through the bone at a 90° | | |

| | | |
|---|---|---|
| angle about 2 cm from the protrusion in the direction of the left leg. Drill until the needle has disappeared into the bone. Then remove the puncture needle. | | |
| | The I.O. access is placed | |
| If access is not successful, a new attempt is made (with a new needle if necessary). | | |
| Now fix the access with the plaster. The plaster includes a small supply tube. Vent this with the prepared infusion. Then connect the infusion and start it. | | |
